# Do climate change adaptation strategies improve farmers’ food security in Tanzania?

**DOI:** 10.1007/s12571-023-01348-6

**Published:** 2023-03-31

**Authors:** Girma Gezimu Gebre, Yuichiro Amekawa, Asmiro Abeje Fikadu, Dil Bahadur Rahut

**Affiliations:** 1 The Japan Society for the Promotion of Science (JSPS) Postdoctoral Research Fellowship Program, Ritsumeikan University, Kyoto 603-8577, Japan; 2 Faculty of Environment, Gender and Development Studies, Hawassa University, Hawassa, Ethiopia; 3 College of International Relations, Ritsumeikan University, Kyoto, Japan; 4 Department of Agricultural Economics, Debre Tabor University, Debre Tabor, Ethiopia; 5 Department of Agricultural and Resource Economics, Graduate School of Bioresource and Bioenvironmental Sciences, Kyushu University, Fukuoka, Japan; 6 Asian Development Bank Institute, Tokyo, Japan; 7 International Maize and Wheat Improvement Center, Mexico, Mexico

**Keywords:** Climate risk adaptation strategies, Household food security, Endogenous switching regression, Multivariate probit, Tanzania

## Abstract

The damaging effects of changing climate on farm-household food security are steadily increasing in sub-Saharan Africa. Adaptation strategies are important for agrarian households to reduce the adverse effects on their food security. This study employed multivariate probit and endogenous switching regression models to analyze the determinants of farm households’ choice of climate-change adaptation strategies, such as the cultivation of early maturing crops, early planting, growing drought-tolerant maize varieties, using precautionary savings, practicing income diversification, and sale of assets, and their effects on household food security in Tanzania. Information on expected rainfall and temperatures, early warning systems, previous droughts, delays in the onset of the rainy season, sex and age of the farmer, educational level, farming experience, family size, total farmland holding, number of livestock owned, contact with extension agents, and access to credit services were all found to influence decisions by farm households to use strategies of adaptation to climate change. Overall, the adaptation of farm households to climate change increased their food security status. An analysis of “adapter” and “non-adapter” farm households showed that the effect of adaptation on food security was smaller for households that adapted than for households that did not. Thus, we recommend that further effective adaptation strategies such as planting drought-resistant crops, changing planting dates, planting early maturing crops, and practicing income diversification be developed and used, particularly for the most vulnerable farm households, to mitigate the adverse impacts of climate change on their food security.

## 1 Introduction

Agricultural production is vital to ensure global food security, but climate change poses a significant and growing threat to the productivity of global agricultural systems and their associated food security (Intergovernmental Panel on Climate Change [IPCC], 2014; Shahbaz et al., 2021). Climate change also has negative consequences for other ecosystems and their services to humans (De Pinto et al., 2019; IPCC, 2022). Climate change disproportionately affects vulnerable populations living in agricultural communities in developing countries (Amole & Ayantunde, 2019; Rahut & Ali, 2017), and is expected to affect more people in more areas in the future (De Pinto et al., 2019). The worst-hit areas will include sub-Saharan Africa (SSA) (Akinnagbe & Irohibe, 2014; Hadebe et al., 2016; Zakari et al., 2022), where food security assurance remains a major challenge and human populations are highly vulnerable to climatic and other shocks (Drammeh et al., 2019; Gebre & Rahut, 2021; Kabubo-Mariara & Mulwa, 2019; Ndiritu & Muricho, 2021).

In recent decades, climate change has intensified in SSA (Franklin et al., 2021; IPCC, 2007), posing a threat to the sustainability of food production by small-scale rural communities that are dependent on rainfed agriculture for their livelihood (Atube et al., 2021; Di Falco et al., 2011; Fadina & Dominique, 2018; Nelson et al., 2009; Zakari et al., 2022). The IPCC (2007) and the International Food Policy Research Institute (IFPRI, 2010) predict that, by 2050, crop productivity in SSA will have declined by 5% for maize, 14% for rice, and 22% for wheat, pushing many already vulnerable people who depend on agriculture for their livelihoods deeper into poverty and food insecurity. IPCC and IFPRI also predict that by 2050, food availability in SSA will decrease by 500 cal per person (a 21% decline), while over 10 million malnourished children will be added, reaching a total of 52 million. The World Bank reported that, without concrete climate and development actions, up to 86 million people could become climate migrants within their own countries in the SSA region by 2050 (World Bank, 2021). Accordingly, individuals, families, and even whole communities could be displaced and forced to seek more viable and less vulnerable places to live (Rigaud et al., 2018). Of them, 38.5 million would come from the Lake Victoria basin countries of Kenya, Tanzania, and Uganda in East Africa (World Bank, 2021).

The adverse effects of climate change in East African countries are severe because of the interaction of multiple factors, including high human population growth, extreme poverty, inadequate infrastructure, overdependence on rainfed agriculture, poor availability and quality of meteorological data, and knowledge gaps (Agidew & Singh, 2018; Brüssow et al., 2017, 2019; Drammeh et al., 2019; Gebre & Rahut, 2021; IPCC, 2007, 2014; Kabubo-Mariara & Mulwa, 2019; Ndiritu & Muricho, 2021). This makes it imperative for the region to explore adequate adaptation and mitigation strategies to offset the current and future adverse impacts of a changing climate. An adaptative approach to climate change production and livelihood (including the planting of drought-tolerant crops, changes in sowing dates, managing water resources, and diversification of incomes) can enable these countries to diversify livelihood sources and become less reliant on sectors that are more susceptible to climate change effects while helping people to improve their capacity to withstand disastrous shocks. Early adaptation can promote development by reducing the risks and costs associated with asset losses from climate-related disasters, minimizing infrastructure repair costs, and creating new opportunities (USAID, 2012; World Bank, 2019).

Various agricultural management techniques are increasingly used to help adapt to climate change. These include the planting of drought-tolerant crops, early planting of crops, crop diversification, rainwater harvesting, development of meteorological forecasting capabilities, market responses (e.g., income diversification and credit programs), and improvements in agricultural markets and information provision (De Falco et al., 2011; Ali & Erenstein, 2017; Brüssow et al., 2017; IPCC, 2018; Fadina & Dominique, 2018; Kabubo-Mariara & Mulwa, 2019; De Pinto et al., 2019; Bairagi et al., 2020; Kogo et al., 2020; Aryal et al., 2020; Franklin et al., 2021; Atube et al., 2021; Gebre & Rahut, 2021; Shahbaz et al., 2022).

Tanzania has experienced severe weather events, such as droughts, floods, and windstorms, which impose high economic costs (Kijazi & Reason, 2009; Future Climate for Africa, 2017). Economic losses in the agricultural sector related to climate change are estimated to be approximately US$200 million annually (International Center for Tropical Agriculture [CIAT] & World Bank, 2017). The projected impacts of climate change in Tanzania are likely to rise, with significant consequences for its key socioeconomic sectors that could hamper future development. Furthermore, climate change directly and indirectly influences food security, with adverse implications on the availability and accessibility of food in the country (IPCC, 2014). In recognition of this, the government of Tanzania has encouraged the use of climate change adaptation strategies such as rainwater harvesting and irrigation, the use of drought-resistant crops, income diversification, crop income diversification, and various soil management strategies such as mulching and terracing, crop rotation, intercropping, and tree planting, as recommended by several studies including Arndt et al. (2012), Kahimba et al. (2015), Shikuku et al., 2017, IPCC (2018); Komba and Muchapondwa (2018); Kabubo-Mariara and Mulwa (2019); Brüssow et al. (2017, 2019) and Ogada and et al. (2021). Effective engagement in these strategies will require overcoming existing key barriers pertaining to the availability and accessibility of useful information as well as the state’s limited capacity to accurately predict future changes in climate and assess their sectoral impacts (USAID, 2012).

Numerous studies are now available on the factors affecting the adoption of climate change adaptation strategies in various developing countries (see Brüssow et al., 2019; Shahbaz et al., 2022). Nevertheless, studies on the impact of these adopted strategies on the food security of rural households are scarce in developing countries, including Tanzania. This study contributes to the literature on climate change and agriculture by providing a micro-perspective on the effects of climate change on people’s adaptation and food security in Tanzania. Specifically, we investigated the determinants of smallholder farm households’ choice of multiple climate change adaptation strategies in Tanzania using multivariate probit regression. We also assessed the impact of the use of these adaptation strategies on household food security using an endogenous switching regression.

The rest of the paper is structured as follows: Section 2 discusses the conceptual framework and analytical methods; Section 3 describes the study area, nature of the data, and sampling procedures; Section 4 presents the results and discussion; and Section 5 concludes the paper with a note on policy implications.

## 2 Study area, data, and sampling procedures

### 2.1 Study area

**Fig. 1:**
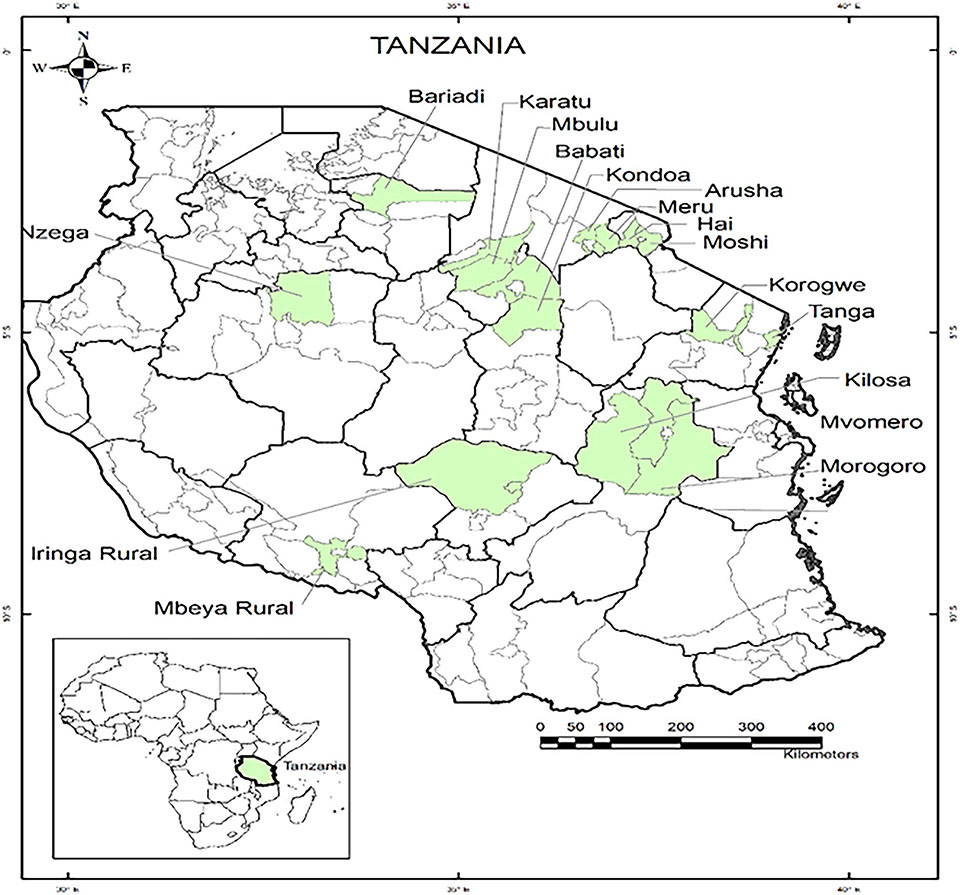
Map of the study areas in Tanzania for the Stress-Tolerant Maize for Africa (STMA) project. The green shaded areas were the survey sites

The study was based on a set of household survey data collected in December 2018 through the Stress-Tolerant Maize for Africa (STMA) project. The STMA project aimed to help farmers mitigate the combined effects of multiple stressors, such as drought, heat, poor soil fertility, and diseases that affect maize production and farming, to improve the food security and livelihoods of smallholder farmers in Tanzania. The sampling procedure to identify study areas and respondent households was designed by researchers from the International Wheat and Maize Research Center (CIMMYT) in collaboration with the agricultural personnel of regional and district-level governments in Tanzania. The survey involved 720 households in 39 villages across 17 districts and 10 regions (see Fig. 1). Surveyed areas included Morogoro region (Kilosa, Morogoro rural, and Mvomero districts), Iringa (Iringa district), Mbeya (Mbeya district), Tabora (Nzega district), Manyara (Mbulu and Babati districts), Simiyu (Bariadi district), Tanga (Korogwe and Tanga districts), Dodoma (Kondoa district), Arusha (Karatu, Meru, and Arusha districts), and Kilimanjaro regions (Moshi rural and Hai districts).

### 2.2 Data and sampling procedures

A multistage sampling technique combining purposive and random sampling was used. The first stage selected regions and the second, districts. A third stage involved selecting villages using probabilities proportional to the sample size design. The fourth and final stages involved a random sampling of households in each village. In each household, the person most responsible for household food production (i.e., the head of household) was selected for face-to-face interviews at home or on the farm. A semi-structured questionnaire was designed and tested to capture the survey data. Trained and experienced enumerators administered the questionnaire under the supervision of researchers from CIMMYT. The collected data included detailed information on households’ socioeconomic and demographic characteristics, asset holdings, social networks, food security, climate change, and adaptation strategies, as well as relevant information on institutions (for more details, see the questionnaire provided as supplementary material).

## 3 Conceptual framework and analytical methods

### 3.1 Conceptual framework

Unstable climatic conditions can adversely affect agricultural production and, hence, the food security of farm households (Di Falco et al., 2011; IPCC, 2014; Brüssow et al., 2017; Teklewold et al., 2017; IPCC, 2018; Eitzinger et al., 2018; Sonko et al., 2020; Gebre & Rahut, 2021). In our study we employed a standard definition of food security as a situation that exists when all people, always have physical, social, and economic access to sufficient, safe, and nutritious food that meets their dietary needs and preferences for an active and healthy life (FAO, 1996). Farm households may decide to adopt different adaptation strategies to reduce the adverse impacts of climate change on food security (Di Falco et al., 2011; Amare & Simane, 2017; IPCC, 2018; Haq et al., 2021; Ojo et al., 2021). Decisions by farm householders to adopt various adaptation strategies are affected by their demographic, socioeconomic, agronomic, and other natural forms of capital (Ali & Erenstein, 2017; Aryal et al., 2020; Brüssow et al., 2017; Gebre & Rahut, 2021).

### 3.2 Analytical methods

Other studies have shown that farm households in Tanzania are more likely to adopt multiple adaptation strategies simultaneously to reduce the effects of climate change hazards on food security (Brüssow et al., 2017; Future Climate for Africa, 2017; Shikuku et al., 2017; Ogada, et al., 2021). The decision to adopt one strategy can influence the adoption of other strategies (Ogada, et al., 2021). In these cases, applying a multivariate probit model yields unbiased and efficient estimates (Greene, 2019; Wooldridge, 2012). Thus, we employed a multivariate probit model to examine the determinants of their choice of climate change adaptation strategies, including growing early-maturing crops, early planting of crops, using drought-tolerant maize varieties, income diversification (e.g., finding off-farm income sources), use of precautionary savings, and selling assets (e.g., livestock).^1^ The multivariate probit is equipped with the effect of a set of regressors for each adaptation strategy simultaneously, while allowing free correlation among the unexamined factors (Lin et al., 2005).

Given a set of adaptation strategies, we assumed that a risk-averse farmer (S) will choose an adaptation strategy ($F_{i1}$) that yields a higher utility (Y) than the alternative adaptation strategy ($F_{i2}$), as shown in Eq. (1):

(1) $$U[S(Y)]=U[S(F_{i1})]>U[S(F_{i2})]$$

Since the utility cannot be observed, it is represented as a function of observable components (Ojo et al., 2021), as expressed in Eq. (2):

(2) $$Y_{rf}^{*}=\alpha_{f}X_{rf}+\beta_{f}V_{rf}+\varepsilon_{f}\;\text{where}\;(f=1,\ldots \;..m)$$

$$Y_{\text{rf}}=1\;\text{if}\;Y_{\text{rf}}^{*}>0\;\text{and}\;0\;\text{if otherwise}$$

where $Y_{rf}^{*}$ represents the latent variable indicating the unobserved outcome, and is associated with $f^{th}$, which represents climate change adaptation strategies. The $Y_{rf}$ denotes the binary dependent variable, and (f=1,….m) represents the strategies adopted by farm households in the study area (i.e., the use of drought-tolerant maize varieties, income diversification, early planting, precautionary savings, selling assets such as livestock, and cultivation of early-maturing crops). The farm household was assigned a value of 1 if any adaptation strategy is chosen, and 0 otherwise. $X_{rf}$ is the vector of explanatory variables in the model. $\alpha_{f}$ and $\beta_{f}$ represent the parameters to be estimated. The error term $\varepsilon_{f}$ in the model has multivariate normal distributions with zero conditional mean, unitary variance, and an n × n correlation matrix (Mulwa et al., 2017). The resulting covariance matrix (ϖ) is given by Eq. (3):

(3) $$\varpi =\left[\begin{array}{cccccc} 1 & \rho 12 & \rho 13 & \rho 14 & \rho 15 & \rho 16\\ \rho 21 & 1 & \rho 23 & \rho 24 & \rho 25 & \rho 26\\ \rho 31 & \rho 32 & 1 & \rho 34 & \rho 35 & \rho 36\\ \rho 41 & \rho 42 & \rho 43 & 1 & \rho 45 & \rho 46\\ \rho 51 & \rho 52 & \rho 53 & \rho 54 & 1 & \rho 56\\ \rho 61 & \rho 62 & \rho 63 & \rho 64 & \rho 65 & 1 \end{array}\right]$$

where ρ denotes the pairwise correlation coefficient of error terms corresponding to any two climate adaptation strategies. If the off-diagonal elements in the covariance matrix become nonzero, then applying a multivariate probit instead of a univariate probit for each individual climate adaptation strategy is justifiable.

Second, an endogenous switching regression was employed to estimate the impact of climate change adaptation strategies on household food security in Tanzania. The decision of farm householders to adopt climate change adaptation measures is voluntary and may be based on the household’s self-selection.^2^ Farmers who adopted certain adaptation strategies might have systematically different characteristics from those who did not, and the former may have decided to adapt based on expected benefits. Evaluating the effect of an adaptation measure on food security by estimating a single outcome equation using a dummy adaptation variable as one of the explanatory variables will yield biased estimates. The use of endogenous switching regression can ensure heterogeneity in farmers’ adoption decisions by considering the unobservable characteristics of surveyed farm households. Endogenous switching regression estimation significantly reduces selection bias by controlling for both observed and unobserved differences between treatment groups by constructing counterfactuals (Kassie et al., 2014). Numerous studies have used endogenous switching regression to conduct impact studies, including Di Falco et al. (2011), Asfaw et al. (2012), Shiferaw et al. (2014), Khonje et al. (2015) and Ojo et al. (2021).

As adaptation to climate change is also potentially endogenous, we employed an endogenous switching regression following Di Falco et al. (2011), Asfaw et al. (2012), Khonje et al. (2015), Ojo et al. (2021), and Ndiritu and Muricho (2021). The endogenous switching regression is a two-stage procedure. In the first stage, a household was assumed to adopt at least one climate adaptation strategy if the utility from the adaptation ($U_{i1}$) is higher than the utility from non-adaptation ($U_{i0}$), that is, if the utility derived from the adoption ($U^{*}$) is greater than 0:

(4) $$U\;*=U_{i1}-U_{i0}>0$$

Since this utility is unobservable, the farmers’ decision to adopt at least one climate adaptation strategy can be expressed as a function of observable characteristics ($X_{i}$) and the error term ($\varepsilon_{i}$) in the following latent variable model, which is estimated using a binary probit regression:

(5) Ai∗=Ziα+ηi;withAi={1ifAi∗>00otherwise}

where $A_{i}^{*}$ is the latent (unobserved) binary variable indicator of climate change adaptation; $A_{i}$ is the observed binary indicator variable of climate change adaptation, which is equal to 1 if the farm household has adopted at least one climate adaptation strategy, and 0 if it has not; α is a vector of parameters to be estimated; $Z_{i}$ is the vector of explanatory variables that determine climate change adaptation; and $\eta_{i}$ is the error term normally distributed with zero mean and constant standard variance.

Based on past studies, we hypothesized that adaptation to climate change would have a significant positive impact on the food security of surveyed households. In our study, we employed the Household Food Insecurity Access Scale (HFIAS) indicators to construct the food security measure/cut-off point that was developed by the United States Agency for International Development (USAID) Food and Nutrition Technical Assistant (FANTA) project. The HFIAS indicator assumes that the experience of food (in)security causes predictable reactions and responses that can be captured and quantified through a survey and summarized on a scale (Coates et al., 2007; Headey & Ecker, 2012). Using the HFIAS approach, respondents were asked nine questions related to food insecurity and the frequency of its occurrence over a four-week recall period (Table 1). The “occurrence” questions were used to measure the severity of food insecurity, and the “frequency-of-occurrence” questions were asked as a follow-up to each occurrence question to determine how often the condition occurred. For instance, if the respondent answered “yes” to an occurrence question, a frequency-of-occurrence question was asked to determine whether the condition happened rarely (once or twice), sometimes (three to ten times) or often (more than ten times) in the past four weeks (Coates et al., 2007). The HFIAS approach yields information on food and security at the household level (Gebre & Rahut, 2021).

The HFIAS indicator categorizes households into four levels: those with food security, mild food insecurity, moderate food insecurity, and severe food insecurity. Following Coates et al. (2007), the operational definitions of household food and security used in this study were as follows. *Food-secure* describes a situation where a household experiences none of the food insecurity conditions, or just experiences anxiety, but only rarely. *Mildly food-insecure* describes the situation where a household worries about not having enough food sometimes or often, or is unable to eat preferred foods, eat a more monotonous diet than desired, and/or consume some foods considered undesirable, but only rarely; however, the household does not cut back on quantity, nor does it experience any of the three most severe conditions: running out of food, going to bed hungry, or going a whole day and night without eating. *Moderately food-insecure* describes a situation where a household sacrifices quality more frequently by eating a monotonous diet or undesirable foods sometimes or often, and/or has started to cut back on quantity by reducing the size of meals or the number of meals, rarely or occasionally. *Severely food-insecure* describes a situation where a household has graduated to cut back on meal size or the number of meals often, and/or experiences any of the three most severe conditions (running out of food, going to bed hungry, or going a whole day and night without eating), even as infrequently as rarely.

In this study, the household food (in)security access category variable was calculated using the assigned codes for the degree of food security states in which they fell. Accordingly, four categories of food security status were created sequentially (1 = food-secure, 2 = mildly food-insecure, 3 = moderately food-insecure, and 4 = severely food-insecure) to ensure that households were classified according to their most severe food security response. Due to the small sample size, we merged three food-insecure statuses (mildly, moderately, and severely) into “food-insecure” and the rest into “food-secure” categories. Thus, the dependent variable (outcome variable) was binary, with “one” assigned to a food-secure household and “zero” to a food-insecure household.

Subsequently, a two-stage endogenous switching regression model was established (Asfaw et al., 2012; Ayuya et al., 2015; De Falco et al., 2011; Khonje et al., 2015; Ojo et al., 2021). In the first stage, we modelled a farm household decision (adoption or non-adoption) in Eq. (5), based on the estimation using a binary probit regression. In the second stage, a binary probit model was adopted. This model applies a selectivity correction to examine the relationship between the outcome variables that are conditional on farmers’ adaptation decisions. The two outcome equations conditional on adaptation are defined as follows:

(6) $$Regime\;1\;:\;Y_{1i}=X_{1i}\beta_{1}+\varepsilon_{1i}\;\;if\;A_{i}=1$$

(7) $$Regime\;2\;:\;Y_{2i}=X_{2i}\beta_{2}+\varepsilon_{2i}\;\;if\;A_{i}=0$$

where $Y_{1i}$ is the food security probability of households that have adapted to climate change, while $Y_{2i}$ is the food security probability of households that have not adapted, $\beta_{1}$ and $\beta_{2}$ are vectors of the parameters to be estimated, $X_{1i}$ and $X_{2i}$ are vectors of explanatory variables, and $\varepsilon_{1i}$ and $\varepsilon_{2i}$ are random disturbance terms. In the endogenous switching regression model, the error terms in Eqs. (5), (6), and (7) are assumed to have a trivariate normal distribution, with a zero mean and covariance matrix ∑, that is, $\left(\eta ,\varepsilon_{1},\varepsilon_{2})∼N(0,\sum \right)$

$$With\;\sum =\left[\begin{array}{ccc} \sigma_{\eta }^{2} & \sigma_{\eta 1} & \sigma_{\eta 2}\\ \sigma_{1\eta } & \sigma_{1}^{2} & \cdot \\ \sigma_{2\eta } & \cdot & \sigma_{2}^{2} \end{array}\right]$$

where $\sigma_{\eta }^{2}$ is the variance of the error term in the selection Eq. (5), which can be assumed to be equal to 1, because the coefficients are estimable only up to a scale factor (see Maddala, 1983, p.223); $\sigma_{1}^{2}$ and $\sigma_{2}^{2}$ are the variances of the error terms in the welfare outcome functions, that is, Eqs. (6) and (7); and $\sigma_{1\eta }$ and $\sigma_{2\eta }$ represent the covariance of $\eta_{i}$, $\varepsilon_{1i}$, and $\varepsilon_{2i}$.

As $Y_{1i}$ and $Y_{2i}$ cannot be observed simultaneously, the covariance between $\varepsilon_{1i}$ and $\varepsilon_{2i}$ is not defined (and is therefore reported as a dot in the covariance matrix (∑) (see Maddala, 1983, p.224; Lokshin & Sajaia, 2004). This type of error structure implies that because the error term of the selection Eq. (5) $\eta_{i}$ is correlated with the error terms of the outcome Eqs. (6) and (7) ($\varepsilon_{1i}$ and $\varepsilon_{2i}$), the expected values of $\varepsilon_{1i}$ and $\varepsilon_{2i}$ conditional on sample selection are nonzero, that is,

$$E[\varepsilon_{1i}\;|\;A_{i}=1]=\sigma_{1\eta }\frac{\phi (Z_{i}\alpha )}{\Phi (Z_{i}\alpha )}=\sigma_{1\eta }\lambda_{1i},\;\text{and}\;\;E[\varepsilon_{2i}\;|\;A_{i}=0]=\sigma_{2\eta }\frac{\phi (Z_{i}\alpha )}{1-\Phi (Z_{i}\alpha )}=\sigma_{2\eta }\lambda_{2i}$$

where ϕ(.) represents the standard normal probability density function, Φ(.) is the standard normal cumulative density function; $\lambda_{1i}=\frac{\phi (Z_{i}\alpha )}{\Phi (Z_{i}\alpha )}$, $\lambda_{2i}=\frac{\phi (Z_{i}\alpha )}{1-\Phi (Z_{i}\alpha )}$, and $\lambda_{1}$, and $\lambda_{2}$ represent the inverse Mills ratios computed from the selection Eq. (5) and will be included in Eqs. (6) and (7) to correct for the selection bias in a two-stage estimation procedure, that is, the endogenous switching regression model (Khonje et al., 2015). If the estimated covariances ${\hat{\sigma }}_{1\eta }$ and ${\hat{\sigma }}_{2\eta }$ are statistically significant, then the decision to adapt to climate change and a household’s food-secure condition are correlated; that is, we find evidence of endogenous switching and thus reject the null hypothesis of the absence of sample selectivity bias. This model is defined as a “switching regression model with endogenous switching” (Maddala & Nelson, 1975).

The full information maximum likelihood (FIML) approach is efficient in estimating endogenous switching regression models (Lee & Trost, 1978). It estimates the selection Eq. (5) and outcome Eqs. (6) and (7) simultaneously (Ayuya et al., 2015; Lokshin & Sajaia, 2011; Ojo et al., 2021).

### 3.3 Conditional expectations, treatment, and heterogeneity effects

The endogenous switching regression model can be used to compare the actual expected food security probability between adapting farm households and those that did not. It also helps to investigate the expected food security probability in hypothetical counterfactual cases where the farm households that reported they adapted are assumed not to have adapted, and where the farm households that did not adapt are considered to have adapted. Therefore, we used Eq. (6) to estimate the actual food security probability among adapters to climate change and then used the coefficients from that equation to compute the mean counterfactual food security probability among farm households that did not adapt to climate change. Similarly, we used Eq. (7) to estimate the actual food security probability among households that did not adapt to climate change and then used the derived coefficients to compute the mean counterfactual food security probability for climate change adapters. The actual and counterfactual food security probabilities among adapter and non-adapter farm households were computed in an endogenous switching regression framework as follows:

Actual scenarios:

(8a) $$\text{Adapting households}:E(Y_{1i}|Ai=1)=X_{1i}\beta_{1}+\sigma_{1\eta }\lambda_{1i}$$

(8b) $$\text{Non-adapting households}:E(Y_{2i}|Ai=0)=X_{2i}\beta_{2}+\sigma_{2\eta }\lambda_{2i}$$

Counterfactual scenarios:

(8c) $$\text{Adapting households had they not adapted}:\;\;E(Y_{2i}|Ai=1)=X_{1i}\beta_{2}+\sigma_{2\eta }\lambda_{1i}$$

(8d) $$\text{Non-adapting households had they adapted}:\;\;E(Y_{1i}|Ai=0)=X_{2i}\beta_{1}+\sigma_{1\eta }\lambda_{2i}$$

We applied these conditional expectations and used climate change adaptation as a treatment (TT) to compute the treatment effects among sampled households, as shown in Table 2.

Regarding the endogenous switching regression model to be identified, the $Z_{i}$ variables in Eq. (5) should contain at least one selection instrument variable (Di Falco et al., 2011; Kassie et al., 2014) which significantly affects the selection model (adaptation to climate change) but not the outcome variable (food security) (Ndiritu & Muricho, 2021). Following previous empirical studies (Di Falco et al., 2011; Di Falco & Veronesi, 2013; Ndiritu & Muricho, 2021, and Sarr et al., 2021), we hypothesized that “famers’ access to information on rainfall and temperature,” “early warning,” and “their perceptions of climate change,” particularly regarding their perception of the number of weather extremes (droughts and prolonged dry spells) they had experienced in the 10 years prior to our survey, are factors that directly affect climate change adaptation decisions, rather than affecting household food security. Thus, we used these four factors as part of the explanatory variables in the selection Eq. (5) but excluded them from the subsequent outcome Eqs. (6) and (7). The perceived frequency of climate extremes, such as drought and prolonged dry spells, explains farm households’ adaptation behavior but not the food security outcome. Specifically, Chen and Whalen (2016), Ayanlade et al. (2017), and Ndiritu and Muricho (2021) show that subjective experiences of climate variability and climate change affect farmers’ decisions regarding whether to adapt to climate change. We expected this to be the case for the sampled farm households in the study area. Our exclusion restriction was that farm households’ perception of the number (frequency) of dry spells and droughts does not directly affect the outcome variable (food security), but it does affect indirectly through their climate change adaptation decisions. Thus, unless one’s perception results in a person performing a certain action, that perception alone will not affect the agricultural production outcome, which entails a food security outcome. Similarly, because access to information (e.g., rainfall, temperature, and early warning) directly affects the decision to adapt to climate change, the resultant outcome will affect the food security outcome of agrarian households. However, having access to information on rainfall and temperature alone, without such access leading to climate change adaptations, will not affect farm households’ food security (Ndiritu & Muricho, 2021).

## 4 Results and discussion

### 4.1 Descriptive results

Table 3 presents the descriptive statistics of the variables included in the econometric model estimation. Farm households in the study area reported to have used several measures to adapt to climate change, which can be classified into six major strategies. The most dominant strategy employed by the surveyed farm households was the use of drought-tolerant maize varieties (38%), followed by income diversification (30%), early planting of crops (20%), the use of precautionary savings (12%), and selling assets such as livestock (11%). The results also showed that about 12% of the surveyed households grew early maturing crops, such as improved maize varieties and beans, to mitigate the loss of their production due to climate-related risks. Komba and Muchapondwa (2018) noted that farmers who perceived changes in precipitation and temperature had a higher probability of adopting drought-resistant crops, changing planting dates, and using irrigation to reduce risk from climate change in Tanzania.

Approximately 46% of the surveyed farm households regularly received information on expected rainfall and temperature, while 32% received information from an early warning system.^3^ Between 2008 and 2018, the agricultural production of the surveyed farm households was affected (on average) 1.88 times by drought and 4.5 times by a delay in the arrival of the rainy season. These results indicate that extremely dry weather adversely affected crop production by sampled households in the 10 years prior to this survey. That result is in line with a study by Kijazi and Reason (2009), who noted that the prolonged drought from 1998 to 2005 caused devastating crop failures, livestock losses, and reductions in water reservoir levels, which, in turn, created food shortages and rationing of hydroelectric power and water in Tanzania.

Most surveyed households (86%) were headed by males, with only about 14% headed by females. The average age of the heads of the surveyed households was 52.2 years, with 28 years of farming experience and 6.31 years of education. The average number of household members was 5.74. The majority of farm households in the study area were smallholders with a total average farmland size of 3.3 ha. The average number of livestock owned by the sampled farm households (measured in Tropical Livestock Units)^4^ was 3.21. Approximately 64% of the sampled households had contact with a government extension agent at least once a month. Around 41% of households had access to credit services. Distance to the main market and agricultural development agent was 8.12 km and 3.6 km, respectively. Household membership in agricultural input supply cooperatives and ownership of radio or television were 20% and 66%, respectively.

The study disaggregated surveyed households into adopters/users and non-adopters/users of climate change adaptation strategies, and examined the effect of their climate change adaptation strategies on the food security status of the surveyed households. Approximately 75% of the households surveyed adopted/used at least one adaptation strategy, while the rest (25%) did not employ any measures to cope with climate change-related risks (Table 4). Regarding the food security status of the surveyed households, 48% were in the food-secure category, while the remaining 52% were categorized food-insecure. In the food-insecure category (out of 52%), about 22% were mildly food-secure, while the remaining 24% and 6% were moderately and severely food insecure, respectively. Adopters were more food-secure (51%) compared to non-adopters (25%), with a significant difference at 1% level. Non-adopters were more food-insecure (at 75%, i.e., the sum of mildly (15%), moderately (49%), and severely (11%)) than adopters (at 49%, i.e., the sum of mildly (24%), moderately (19%), and severely (6%)). The differences in all food insecurity categories were statistically significant at the 1% level. These results supported the hypothesis that agricultural households that adopt climate change adaptation strategies are more food secure than those that do not. These results were then rigorously tested using an econometric model.

### 4.2 Econometric results

#### 4.2.1 Determinants for the choice of climate change adaptation strategies

Table 5 presents the correlation matrix of the six adaptation strategies from the multivariate probit estimation. Of the 14 pairwise correlation coefficients of the residuals of climate adaptation strategies, seven were statistically significant, which indicates that the error terms in the selection decisions of multiple climate adaptation strategies are correlated. Of these seven, two had positive coefficients, indicating a positive relationship between the two strategies. First, farm households that plant crops early to adapt to climate change were also more likely to plant early-maturing crops. Second, households that seek to adapt to climate change by using precautionary savings were more likely to grow early maturing crops. The remaining five statistically significant pairs showed a trade-off relationship with negative coefficients. The result of the likelihood ratio test χ^2^ (18) = 70.3095 and prob > χ^2^, *P* > 0.000*** for the independence of the error terms in the different equations indicates that the null hypothesis is rejected. Therefore, in this study we accepted an alternative hypothesis of independence among the different adaptation strategies, justifying the use of the multivariate probit model in the analysis of farm households’ adoption of climate change adaptation strategies. The results also showed that the joint probability of using adaptation strategies was 37%, whereas the probability of not using adaptation strategies was 16%. This indicated that farm households that grow drought-tolerant maize varieties are less likely to adopt other adaptation strategies (see Table 5).

In Table 6 we present the parameter estimates of the multivariate probit model regarding the factors that influence farm households’ choice of adaptation strategy in the study area. All four climate factors examined had a significant effect on the three adaptation strategies, and five demographic or socioeconomic factors had a significant effect on at least three adaptation strategies (sex, family size, education, extension service contact, and credit). Households with access to information on expected rainfall and temperature are more likely to grow drought-tolerant maize varieties, grow early maturing crops and use precautionary savings. Di Falco et al. (2011) reported similar results in their study in Ethiopia, noting that farmers who were informed about climate conditions were more likely to adapt to climate change. Households with access to information on early warning systems are more likely to grow drought-tolerant maize varieties, plant earlier than normal planting time, and use early maturing crop varieties. Households whose crop production is more frequently affected by drought are more likely to grow drought-tolerant maize varieties, use precautionary savings, and sell household assets such as livestock to mitigate the negative impacts of drought. Households whose crop production is more frequently affected by drought due to a delay in the arrival of the rainy season are more likely to grow drought-tolerant maize varieties, diversify income sources, and sell household assets such as livestock. A similar finding was reported by Ndiritu and Muricho (2021) for Kenya.

Our analysis indicated that the sex of the head of household is an important driver of farmers’ choice of climate change adaptation strategies. Compared to female-headed households, male-headed households were more likely to grow drought-tolerant maize varieties and early maturing crops while engaging in income diversification and early planting. Meanwhile, they are less likely to sell household assets such as livestock. This finding is in line with several existing studies such as Deressa et al. (2009) and Aryal et al. (2020). The reason for the male bias might be that male-headed farmers are eager to practice various adaptation strategies to reduce climate change risks. This is because men are considered responsible for much of the agricultural work in the cultural context of the region and, therefore, have greater experience and access to information on various farming and management practices. Another likely reason is that males are more likely to control partial cash crops such as maize for income generation than females.

The age of the head of household was positively associated with growing drought-tolerant maize varieties and using precautionary savings. Maddison (2006) and Ishaya and Abaje (2008) reported similar results about a positive association between age and climate adaptation strategies. However, Denkyirah et al. (2016) and Ojo et al. (2021) reported a negative association between age and adoption of climate change adaptation strategies. According to Ndiritu et al. (2014), aging can be associated with greater energy loss and a more risk-averse tendency. Hence, the positive association between age and growing drought-tolerant maize varieties and that between age and the use of savings may be related to older farmers’ higher preference for risk aversion associated with drought and delay in the arrival of the rainy season in Tanzania. Another reason for the positive association between the age of the head of household and savings is that older people tend to have more money than younger people. A study by Asante et al. (2017) in Ghana noted that older farmers are more likely to have access to productive resources, such as cash in banks, than younger farmers because of their larger savings.

The results also indicated that farm households with more experienced heads are more likely to use precautionary savings and grow early maturing crops. More experienced farmers invest more in the agricultural sector, gain more knowledge and skills through agriculture/climate change adaptation strategies, and the less likely they are to bear risks by producing crops with a long gestation period. Additionally, they are more likely to use precautionary savings to reduce the adverse effects of harsh climatic conditions. Similar findings were reported by Aryal et al. (2020), who noted that more experienced farmers had greater knowledge and more skills and therefore use different adaptation strategies. However, this is not always the case, with Ado et al. (2019) and Ojo et al. (2021) having reported a negative relationship between experience and climate change adaptation strategies.

Family size was positively associated with income diversification, early planting, and growing early maturing crops. The positive and significant coefficient of household size indicates that a farm household with more family members tends to adopt more strategies to minimize climate change-related risks. Other studies have reported similar results (e.g., Kabubo-Mariara & Mulwa, 2019).

The educational level of the farm head of household was positively associated with growing drought-tolerant maize varieties, early maturing crops and income diversification. This suggests that more educated heads of households are more likely to be prepared to address the effects of climate change by adopting innovative farming practices. A positive association between education and adaptation strategies has been found in other studies (see Deressa et al., 2009; Abid et al., 2015; Ali & Erenstein, 2017).

Land size, a major agricultural input and wealth indicator, was positively associated with the growth of drought-tolerant maize varieties and early planting. This result is in line with the generally reported positive association between farm size and technology adoption (Abid et al., 2015; Bryan et al., 2013), coupled with that between farm size and the adoption of climate change adaptation strategies (Ali & Erenstein, 2017; Jamshidi et al., 2020; Kabubo-Mariara & Mulwa, 2019). Farm households with larger landholdings are more likely to have the capacity to try out and invest in climate risk-mitigation strategies.

Livestock ownership is another proxy for farmer wealth. Thus, households with a larger number of livestock are positively associated with wealth factors such as income diversification and selling assets such as livestock. Zhang et al. (2017) noted that selling livestock herd size is a salient strategy for overcoming the adverse effects of climate change. This could be the reason the sampled households sell their livestock as a livelihood strategy to reduce the risks associated with climate change.

We also found that households that have contact with extension agents are more likely to grow drought-tolerant maize varieties and early maturing crops while engaging in early planting (see Table 5). This indicates the critical importance of farm households in accessing relevant information and other resources through extension agents in the study area when seeking to use listed climate-risk-mitigating strategies. In the study area, climate information such as through updated weather forecasts is one of the services provided by extension agents to farm households. This helps significantly increase the likelihood of households adopting listed adaptation strategies. This finding has been often reported elsewhere, such as by Belay et al. (2017) in Ethiopia and Ojo et al. (2021) in South Africa, who affirmed that access to up-to-date weather information enabled farmers to make informed decisions on planting dates and grow early-maturing crops. Households with membership in an agricultural input–supply cooperative were also more likely to grow drought-tolerant maize varieties and plant early maturing crops, as found by Aryal et al. (2020). Access to credit positively affects the financial ability of farm households to invest in growing drought-tolerant maize varieties and early maturing crops. According to Ojo et al. (2019, 2021), farmers’ decisions to adopt adaptation strategies are heavily influenced by financial support that is readily accessible. Similar to the findings of our study, Marie et al. (2020), Aryal et al. (2020), and Ojo and Baiyegunhi (2020) all found that access to credit provides farmers with the financial ability to deal with the transaction costs associated with undertaking various adaptation options.

#### 4.2.2 Determinants of climate change adaptation and household food security

Table 7 presents the estimation of the endogenous switching probit regression based on full information maximum likelihood. The first column reports the estimation by the probit model of food security with no switching and with dummy variables equal to one if the farm household adopted at least one adaptation strategy, and zero otherwise. The second, third, and fourth columns present the estimated coefficients of selection Eq. (5) on the presence or absence of adaptation to climate change and those of the outcome (food security) Eqs. (6) and (7) for farm households that adopted or did not adopt at least one adaptation strategy, respectively.

The correlation coefficients ($\rho_{j}$) of outcome Eqs. (6) and (7) were statistically significant for the correlation between adopters and non-adopters of climate change adaptation strategies. Thus, self-selection occurred in the adoption of climate adaptation strategies, but it might not have the same effect on non-adopters should they choose to adopt. This finding is supported by studies by Lokshin and Sajaia (2004), Abdulai and Huffman (2014), Khanal et al. (2018), and Ojo et al. (2021).

The results of the selection Eq. (5) suggest that the main drivers of farm households’ decision to adopt climate change adaptation measures were access to information on expected rainfall and temperature, information on an early warning system, the number of droughts, and a delay in the arrival of the rainy season in the last ten years prior to the survey (Table 7, Column (2)). These results are consistent with those of Di Falco et al. (2011), Chen and Whalen (2016), Ayanlade et al. (2017), and Ndiritu and Muricho (2021), who showed that farmers’ subjective experiences of climate change influence their decision to adopt an adaptation strategy. Our findings also suggest that male-headed households, age, family size, farming experience, educational level of the head of household, size of the total farmland, number of livestock owned, and contact with extension agents have significantly positive effects on farm householders’ decisions to adopt climate change adaptation measures.

We now turn to the implications of adaptation to climate change for food security. The simplest approach to examine the effect of climate change adaptation on food security is to estimate a probit model of food security (binary dependent variable) that includes a dummy independent variable equal to one if the household adapted, and zero if they did not (Table 7, Column (1)). The results suggested a significant positive relationship between the probability of being food secure and adoption of climate change adaptation strategies. This assumes that adaptation to climate change and food security is endogenous; thus, estimation using the probit model would yield biased and inconsistent estimates. Moreover, probit estimation does not explicitly account for potential structural differences in food security status between adapters and non-adapters. Therefore, the estimation presented in Columns (3) and (4) of Table 7 accounts for endogenous switching in the outcome (food security) function. It is worth noting that four factors had a significant effect on food security for adapters but did not have such an effect on food security for non-adopters. These include the age of the head of household, family size, membership in input supply cooperatives, and access to credit, which had a significantly positive effect on the food security of adapters than non-adapters.

#### 4.2.3 Impact of climate change adaptation on household food security

Table 8 presents the expected conditional probability of food security under the actual (cells a and b) and counterfactual (cells b and d) conditions. The actual expected probability of being food secure by the farm households that adapted was 51%, while it was 25.4% for the group of farm households that did not adapt, indicating that the adapters are about twice as likely as non-adapters to be food secure.

The results of counterfactual case (c) suggest that the probability of being food-secure among farm households that actually adapted is likely to drop significantly by 14% (i.e., a drop from 51 to 37%, a 27.5% decrease) if they did not adapt. On the other hand, the counterfactual case (d) results indicate that the probability of the non-adapters being food-secure could increase by 24.8% (i.e., an increase from 25.4% to 50.2% or about a 97.6% increase) if they had actually adapted to climate change. This result implies that if non-adapter farm households had adapted, they could have had nearly the same food security status as the farm households that had actually adapted. These results confirm that climate change adaptation among the sampled farm households is critical for ensuring household food security in Tanzania. This conclusion is consistent with previous empirical studies in Ethiopia by Di Falco et al. (2011) and in Kenya by Ndiritu and Muricho (2021).

The results on the heterogeneity effect of climate change adaptation on food security suggest that, even if the adapters were not to adapt (case c), their food security probability would still be significantly higher than that of non-adapters (case b). This finding highlights the presence of some critical sources of heterogeneity that make adapter households better off in their food security position than non-adapters, irrespective of climate change. Factors affecting food security are not confined to farmers’ adoption of climate change adaptation strategies, as reported by farmers, but also comprise other non-climatic factors that were not observed in this study (e.g., income diversification would be resorted to to make daily household livelihoods more sustainable in addition to climate change adaptation goals). Furthermore, the observed transitional heterogeneity effect indicates that the impact of climate change on the food security of adapter households is significantly smaller than that of non-adapter households.

#### 4.2.4 Study limitations

A major limitation of this study is related to its focus on the use of cross-sectional data to gauge the effect of farmers’ climate change adaptation on food security. We suggest that future studies on the effect of climate change adaptation on food security should be based on data collected over multiple periods, given that climate change effects vary over time. Second, where the database allows, there is a need to conduct a comparative analysis across regions in Tanzania, as climate change has different effects across regions. Third, this study failed to clearly differentiate farmers’ actions between different goals such as climate change adaptation and meeting other livelihood needs. Accordingly, the model results suggest that the adoption of climate change adaptation strategies as reported by farmers was not the sole explanatory factor for their food security status; an action of similar categories (e.g., engaging in non-farm work for income diversification) that is not perceived by farmers for the goal of climate change adaptation could also influence the level of their food security. Thus, future studies should employ more interaction-based, qualitative research methods, rather than simply relying on a structured questionnaire, to identify more accurate effects on food security.

## 5 Conclusions and policy implications

This study investigated the factors that determine the choice of climate change adaptation strategies by farm householders and their effects on food security in Tanzania. Six major climate change adaptation strategies adopted by the sampled farm households were identified by householders: growing early-maturing crops, using drought-tolerant maize varieties, planting early, diversifying income, using precautionary savings, and selling assets such as livestock.

Econometric analyses revealed that farm households’ choice of adaptation strategies was affected by selected climatic and demographic/socioeconomic factors. Climatic factors included information on expected rainfall and temperature, an early warning system, drought, and a delay in the arrival of the rainy season. These four factors significantly influence farm households’ decisions to adopt various adaptation strategies. The household demographic and socioeconomic factors that were found to have a significant influence on farmers’ choice of adaptation strategies included sex, age, educational level, farming experiences of the head of household, family size, size of total farmland holdings, number of livestock owned, contact with extension agents, and access to credit services. Of these, five had a significant influence on farmers’ adoption of three or more adaptation strategies: sex, family size, education, extension contracts, and credit.

To investigate how measures to adapt to climate change contribute to the food security of farm households, the study disaggregated the surveyed households into adapters and non-adapters. Accordingly, approximately 75% of the surveyed households were identified as adapters and 25% as non-adapters. Regarding farmers’ food security status, 48% of the total surveyed households were classified as food-secure and 52% food-insecure. The findings showed that adapters were more food-secure (51% of them) than non-adapters to climate change (with only 25% food secure). The findings also showed a positive relationship between the probability of being food secure and adopting climate change adaptation strategies. These results support the hypothesis that agricultural households that practice adaptation strategies are more likely to ensure food security than those that do not adopt any adaptation strategies, but given that many households remain food insecure regardless, there is need for additional food security support measures in these communities.

The analysis of the relationship between climate change adaptation and food security for the two different groups, adapters and non-adapters, suggested that even if the adapters were not to adapt, their food security probability would still be higher than that of non-adapters. This result implies that farm households belonging to the adapter group have some unexamined characteristics that would make them more food-secure, even without implementing adaptation strategies to climate change. We found that the impact of adaptation on food security is smaller for farm households that actually adapted than for farm households that did not adapt to the hypothetical counterfactual case they adapted. This finding is important because it suggests that farm households that did not adapt would benefit the most if they were able to engage in adaptation. If farm households that did not adapt had done so, they would have the same food security status as farm households that actually adapted. This demonstrates that effective climate change adaptation based on agricultural and livelihood measures provides saliently beneficial options for the most vulnerable farm households to improve their food security.

Climate change is considered the main threat to the achievement of the Global Sustainable Development Goals (SDG) by 2030. Strengthening adaptive capacity and integrating climate change measures into national policies, strategies, and planning (SDG 13) could help reduce general poverty and hunger and ensure access by all people, in particular, the poor and people in vulnerable situations, including infants, to safe, nutritious, and sufficient food all year round by 2030 (SDGs 1 and 2). Thus, the findings of the present study call for more planning and implementation of policies incorporating effective climate change adaptation strategies into various forms of agricultural extension and farmer capacitation in Tanzania. The timely dissemination of information on expected rainfall, temperature, and an early warning system are adaptation strategies of paramount importance, which could result in an elevated food security status for all households, irrespective of their characteristics. Facilitation of extension and credit services is also important for supporting farmers’ adoption of adaptation strategies. Contact with an extension agent can give farmers an important source of skills and information for adaptation. Improved access to credit services can offer farmers financial resources to purchase improved agricultural inputs, such as drought-resistant crop varieties, and invest in different sources of income diversification activities. Future policies should also focus on building farm household assets, enhancing farmers’ knowledge through educational programs, increasing their access to multiple livelihood opportunities, and helping improve the access to resources of poor people, women, and less experienced farm households.

## Supplementary Material

Supplementary material

## Figures and Tables

**Table 1:** List of occurrence questions for measuring food (in)security in this study

No.	Content of the questions
1	In the past four weeks, did you worry that your household would not have enough food?
2	In the past four weeks, were you or any household member not able to eat the kinds of foods you preferred because of a lack of resources?
3	In the past four weeks, did you or any household member have to eat a limited variety of foods due to a lack of resources?
4	In the past four weeks, did you or any household member have to eat some foods that you really did not want to eat because of a lack of resources to obtain other types of food?
5	In the past four weeks, did you or any household member have to eat a smaller meal than you felt you needed because there was not enough food?
6	In the past four weeks, did you or any household member have to eat fewer meals in a day because there was not enough food?
7	In the past four weeks, was there ever no food to eat of any kind in your household because of a lack of resources to get food?
8	In the past four weeks, did you or any household member go to sleep at night hungry because there was not enough food?
9	In the past four weeks, did you or any household member go a full day and night without eating anything because there was not enough food?

**Table 2:** Conditional expectations, treatment, and heterogeneity effects among the sampled households

Subsamples	Decision stage		Treatment effects
	To adapt	Not to adapt	
Adapters	(8a): $E(Y_{1i}|A_{i}=1)$	(8c): $E(Y_{2i}|A_{i}=1)$	(8a)−(8c)=TT
Non-adapters	(8d): $E(Y_{1i}|A_{i}=0)$	(8b): $E(Y_{2i}|A_{i}=0)$	(8d)−(8b)=TU
Heterogeneity effects	$(8a)-(8d)={\text{BH}}_{1}$	$(8c)-(8b)={\text{BH}}_{2}$	TH = TT – TU

(8a) and (8b) represent observed expected food security probability; (8c) and (8d) represent counterfactual expected food security probability. $A_{i}=1$ if farm households adapted to climate change; $A_{i}=0$ if farm households did not adapt; $Y_{1i}$: the food security probability if farm households adapted; $Y_{2i}$: the food security probability if farm households did not adapt; TT: the effect of the treatment (i.e., adaptation) on the treated (i.e., farm households that adapted); TU: the effect of the treatment (i.e., adaptation) on the untreated (i.e., farm households that did not adapt); ${\text{BH}}_{i}$: the effect of base heterogeneity for farm households that adapted (i=1), and did not adapt (i=2); TH = (TT – TU ), i.e., transitional heterogeneity

**Table 3:** Definitions and summary statistics of variables used in the model

Variables	Definition	Mean
Dependent variables		
Drought-tolerant maize	1 if the household grows drought-tolerant maize varieties, 0 otherwise	0.38
Income diversification	1 if the household diversifies income sources, 0 otherwise	0.30
Early planting	1 if the household practices early planting, 0 otherwise	0.20
Savings	1 if the household uses precautionary savings, 0 otherwise	0.12
Selling assets	1 if the household sells their assets such as livestock, 0 otherwise	0.11
Early maturing crops	1 if the household grows at least an early maturing crop, 0 otherwise	0.12
Decision to adapt	1 if the household decides to adapt at least one adaptation strategy, 0 otherwise	0.75
Food security	1 if the household is food-secure, 0 otherwise	0.48
Independent variables		
*Climatic factors*		
Information (rain/temp.)	1 if the household regularly receives information on expected rainfall and temperature, 0 otherwise	0.46
Early warning	1 if the household receives information on early warning, 0 otherwise	0.32
Drought	Number of times drought-affected agricultural production over the last ten years	1.88
Rainfall delay	Number of times a delay in the arrival of the rainy season affected major agricultural production over the last ten years (2008–2018)	4.50
*Demographic and socioeconomic factors*		
Sex	1 if the head of household is male, 0 otherwise	0.86
Age	Age of the head of household in years	52.20
Family size	Number of household members	5.74
Experience	Farming experience of the head of household in years	28.00
Education	Educational level of the head of household in years	6.31
Farmland	Total farm size owned by the household in hectares	3.30
Livestock	Number of livestock owned by the household in tropical livestock units (TLU)	3.23
Extension contact	1 if the household contacted government extension agent, 0 otherwise	0.64
Agriculture	1 if the head of household’s occupation is agriculture, 0 otherwise	0.93
Credit	1 if the household accessed credit service, 0 otherwise	0.41
Market	Distance to the main market in km	8.12
Daoffice	Distance to the agricultural development agent office in km	3.60
Membership	1 if the household is a member of an agricultural input supply cooperative	0.20
Owned radio or TV	1 if the household currently owns a radio or television, 0 otherwise	0.66

**Table 4:** Household food security status of adopters and non-adopters

Food security status	Total (*N* = 720)	Adopters (*N* = 540)	Non-adopters (*N* = 180)	Test difference (χ^2^)
Food-secure	48%	51%	25%	***
Mildly food-insecure	22%	24%	15%	***
Moderately food-insecure	24%	19%	49%	***
Severely food-insecure	6%	6%	11%	***

*** denotes the differences are statistically significant at 1% significance level (*p* value = 0.000)

**Table 5:** The correlation of error terms of the selected climate adaptation strategies

Correlation pairs	Coefficient (Standard error)
ρ21 (grow drought tolerant maize varieties and income diversification)	−0.327 (0.059)***
ρ31 (grow drought tolerant maize varieties and early planting)	−0.240 (0.066)***
ρ41 (grow drought tolerant maize varieties and use precautionary savings)	−0.150 (0.078)*
ρ51 (grow drought tolerant maize varieties and sell assets)	−0.105 (0.078)*
ρ61 (grow drought tolerant maize varieties and grow early maturing crops)	−0.091 (0.774)
ρ32 (income diversification and early planting)	−0.069 (0.06)
ρ42 (income diversification and use of precautionary savings)	−0.053 (0.0824)
ρ52 (income diversification and sell assets)	0.024 (0.079)
ρ62 (income diversification and grow early maturing crops)	0.004 (0.077)
ρ43 (early planting and use precautionary savings)	−0.027 (0.088)
ρ53 (early planting and sell assets)	0.075 (0.081)
ρ63 (early planting and grow early maturing crops)	0.143 (0.081)***
ρ54 (use precautionary savings and selling assets)	−0.081 (0.096)**
ρ64 (use precautionary savings and grow early maturing crops)	0.263 (0.083)***
Joint probability of success	0.370 (0.291)**
Joint probability of failure	0.160 (0.241)

Likelihood ratio test of ρ21=ρ31=ρ41=ρ51=ρ61=ρ32=ρ42=ρ52=ρ62=ρ43=ρ53=ρ63=ρ54=ρ64=ρ65=0 χ^2^ (18) = 70.3095 and prob > χ^2^ = 0.000

***, ** and * are statistically significant at 1%, 5% and 10% significance level, respectively. Standard error in the parentheses

**Table 6:** Multivariate probit estimates for the factors associated with the choice of climate change adaptation strategies

Variables	Climate change adaptation strategies					
	Grow drought- tolerant maize varieties	Income diversification	Early planting	Precautionary savings	Sell assets (e.g., livestock)	Grow early maturing crops
*Climatic factors*						
Information (rain/temp.)	0.607 (0.149)***	−0.104 (0.145)	0.011 (0.166)	0.026 (0.231)***	−0.004 (0.191)	0.019 (0.190)**
Early warning	0.207 (0.154)***	0.072 (0.110)	0.421 (0.304)**	0.132 (0.460)	0.208 (0.105)	0.814 (0.925)***
Drought	0.734 (0.487)***	0.921 (1.012)	0.550 (0.841)	0.303 (0611)**	0.472 (0.702)***	0.203 (0.305)
Rainfall delay	0.502 (0.812)***	0.502 (834)***	0.110 (0.020)	0.406 (0.701)	0.002 (104)**	0.573 (0.204)
*Demographic and socioeconomic*						
Sex	0.799 (0.207)***	0.693 (0.194)***	0.881 (0.284)***	0.555 (0.978)	−0.078 (0.066)**	0.272 (0.166)***
Age	0.014 (0.083)*	0.007 (0.008)	−0.004 (0.009)	0.029 (0.013)**	0.010 (0.009)	−0.009 (0.011)
Family size	−0.0263 (0.027)	0.063 (0.021)***	0.070 (0.023)***	−0.008 (0.027)	0.005 (0.026)	0.062 (0.025)**
Experience	0.003 (0.006)	−0.007 (0.005)	0.0004 (0.006)	0.008 (0.007)***	−0.004 (0.007)	0.003 (0.007)***
Education	0.172 (0.019)***	0.033 (0.016)**	−0.005 (0.018)	0.050 (0.207)	0.013 (0.020)	0.057 (0.020)***
Agriculture	0.446 (0.220)	−0.261 (0.196)	0.277 (0.239)	−0.026 (0.242)	0.823 (0.355)	0.361 (0.303)
Market	0.002 (0.008)	−0.004 (0.008)	−0.006 (0.009)	0.008 (0.014)	−0.019 (0.011)	−0.006 (0.010)
DAoffice	−0.043 (0.023)	0.002 (0.020)	−0.034 (0.024)	−0.014 (0.029)	0.028 (0.026)	0.016 (0.026)
Farmland	0.234 (0.080)***	0.095 (0.077)	0.158 (0.084)**	0.077 (0.099)	0.178 (0.098)	−0.007 (0.099)
Livestock	0.003 (0.065)	0.010 (0.066)**	−0.030 (0.069)	0.052 (0.073)	0.125 (0.072)***	0.011 (0.080)
Extension Contact	0.010 (0.162)**	0.275 (0.156)	0.193 (0.183)**	0.591 (0.244)	0.055 (0.212)	0.056 (0.205)***
Owned radio or TV	−0.054 (0.132)	0.077 (0.1244)	0.291 (0.143)	−0.004 (0.162)	0.622 (0.181)	0.070 (0.158)
Membership	0.194 (0.115)**	0.147 (0.10)	0.019 (0.120)	−0.132 (0.149)	−0.097 (0.137)	0.332 (0.155)**
Credit	0.414 (0.121)***	−0.053 (0.116)	0.249 (0.126)**	0.209 (0.072)	0.101 (0.146)	0.038 (0.144)***
_constant	−1.467 (1.520)***	−2.7191 (1.457)	−1.961 (1.565)***	−2.5847 (1.979)	−3.621 (2.768)**	−3.344 (2.659)***
Numbers of observation	720					
Log-likelihood	−1769.6261					
Wald χ^2^ (96)	401.97					
Prob > χ^2^	0.0000					

The dependent variable is adaptation strategy choices

***, ** and * are statistically significant at 1%, 5%, and 10% significance levels, respectively. Standard errors are indicated in the parentheses

**Table 7:** Full information maximum likelihood estimates of the endogenous switching probit model

Model	(1)	(2)	(3)	(4)
	Probit	Endogenous switching probit model		
		Selection equation	Adaptation = 1 (farm households that adapted to climate change)	Adaptation = 0 (farm households that did not adapt to climate change)
Variables	Food security	Adaptation (1/0)	Food security	Food security
Adaptation (1/0)	0.602 (0.204)***			
*Climate factors*				
Information (rain/temp)		0. 307 (0.121)**		
Early warning		0.599 (0.427)**		
Drought		0.501 (0.220)***		
Rainfall delay		0.045 (0.032)***		
*Demographic and socioeconomic factors*				
Sex	0.392 (0.194)**	0.813 (0.149)*	0.301 (0.045)***	−0.879 (0.531)**
Age	0.028 (0.091)	0.062 (0.026)**	0.014 (0.011)**	−0.061 (0.051)
Family size	0.021 (0.043)	0.005 (0.003)**	0.001 (0.001)**	−0.005 (0.005)
Farming experience	0.550 (0.281)	0.001 (0.006)*	0.001 (0.002)	−0.018 (0.011)
Education level	0.023 (0.047)**	0.012 (0.019)**	0.003 (0.007)***	−0.020 (0.036)**
Agriculture	0.071 (0.092)	0.129 (0.221)	0.108 (0.086)	−0.239 (0.422)
Market	−0.685 (0.993)	0.051 (0.023)	0.006 (0.009)	−0.009 (0.046)
DA office	−0.717 (0.800)	0.007 (0.008)	−0.003 (0.003)	0.024 (0.016)
Farmland size	0.032 (0.015)***	0.023 (0.023)*	0.009 (0.009)***	−0.039 (0.039)**
Livestock	0.153 (0.091)**	0.033 (0.012)**	0.005 (0.005)***	−0.008 (0.024)**
Extension contact	0.060 (0.407)**	0.043 (0.024)***	0.061 (0.430)**	−0.056 (0.077)***
Owned radio or TV	0.410 (0.148)	−0.212 (0.252)	0.303 (0.701)	0.901 (0.341)
Membership	0.322 (0455)**	0.147 (0.117)	0.407 (0.322)**	−0.045 (0.081)
Credit	0.087 (0.013)**	0.034 (0.041)	0.078 (0.022)***	−0.121 (0.220)
Constant	−1.173 (1.557)	−1.816 (0.743)**	1.457 (1.296)***	2.123 (1.218)*
$\sigma_{i}$			172.902 (130.529)***	1143.710 (1116.095)**
$\rho_{j}$			−911.667 (1203.103)***	−1024.710 (1036.773)**
Prob > χ^2^	0.000***			
Log-likelihood	−826.14714			
LR test of indep. eqns.:	55.29			
Pseudo R2	0.432	0.527	0.411	0.341
Observation	720	720	540	180

Standard error in parentheses; $\sigma_{i}$ represents the square root of the variance of the error terms $\varepsilon_{ji}$ in outcome Eqs. (6) and (7), respectively. $\rho_{j}$ denotes the correlation coefficient between the error term $\eta_{i}$ of the selection Eq. (5) and error term $\varepsilon_{ji}$ of the outcome Eqs. (6) and (7), respectively

***, **, and * indicate statistical significance at the 1%, 5%, and 10% significance levels, respectively

**Table 8:** Average expected food security status, treatment, and heterogeneity effects

Sub-samples	Decision stage		
	To adapt	Not to adapt	Treatment effects
Households that adapted	(a) 0.510 (0.031	(c) 0.370 (0.017)	TT = 0.140 (0.069)**
Households that did not adapt	(d) 0.502 (0.038)	(b) 0.254 (0.028)	TU = 0.248 (0.084)***
Heterogeneity effects	a–d = 0.008 (0.011)	c–b = 0.116 (0.048)**	TH = −0.108 (0.015)**

(a) and (b) denote the expected household food security status observed in the sample, while (c) and (d) show their counterfactual expected value; standard errors in parentheses

*** and ** are statistically significant at the 1% and 5% significance levels, respectively

## Footnotes

1 The questionnaire used for the collection of data for this study included 12 types of climate adaptation strategies obtained from the participating farmers: (1) growing drought tolerant maize varieties, (2) growing early maturing crops, (3) income diversification (e.g., engaging off-farm work), (4) crop diversification (polyculture or intercropping), (5) early planting, (6) selling livestock, (7) precautionary savings, (8) irrigation, (9) moving farmland to wetland areas, (10) minimum tillage, (11) stone and soil bunds/terracing, and (12) borrowing money. However, for the analysis, we used the six major adaptation strategies based on the number of responses in the samples, as we did not obtain a sufficient number of responses for the rest of the six adaptation strategies.

2 In this study, an “adopter/adapter” is defined as a farm household that reported use of at least one climate change adaptation strategy to reduce the adverse effects of climate hazards, while a “non-adopter/non-adapter” is defined as a farm household that did not use any climate change adaptation strategies.

3 This is an adaptive measure for climate change, using integrated communication systems to help communities prepare for hazardous climate-related events. A successful early warning system can save lives and jobs, land and infrastructure, and supports long-term sustainability.

4 “Tropical Livestock Units” refers to the number of livestock converted to a common unit (Benoit & Veysset, 2021). Conversion factors are: cattle = 0.7, sheep = 0.1, goats = 0.1, pigs = 0.2 and chicken = 0.01.
