# Supplementary material for: Do climate change adaptation strategies improve farmers’ food security in Tanzania?
Source: Food Secur. Author manuscript; Available in PMC 2025 Jan 16. (PMC11737591; doi:10.1007/s12571-023-01348-6)
Supplement: Supplementary material [file NIHMS1946348-supplement-Supplementary_material.docx]

**STMA EARLY ADOPTION SURVEY 2018**

**HOUSEHOLD QUESTIONNAIRE**

**TANZANIA VERSION**

***Enumerator: For this questionnaire you are to interview the HOUSEHOLD HEAD in ALL cases. If the household head is very elderly or away most of the year, then you can ask if another household member knowledgeable of maize farming can sit in on the interview to ASSIST the HOUSEHOLD HEAD. When you first approach the household and introduce yourself you should state your interest to talk with the household head. If the household head is not available, then make an appointment for the interview. If he or she will not be available for a few days, then you should ask your supervisor for a replacement household.***

# CONSENT STATEMENT

***Enumerator, please read in FULL:***

Hello, my name is **___________________**. I am working with CIMMYT on a study about farmer uptake of improved maize varieties in Tanzania. **I would like to ask you** some questions related to your maize production, use of inputs, and general characteristics of your household. The **information you provide** will be used to document the adoption of improved maize varieties in this region, and identify factors that need to be addressed to increase the benefits for farmers from adoption. This interview **will take** approximately **2 hours.** Your participation is **voluntary.** Your **refusal to participate** or to withdraw from the study **carries no penalty** or loss of any benefits**. All information** provided by you will be **kept confidential.** Your **privacy will be protected** to the maximum extent allowable by law. If you have any questions, you can ask me or contact Mariam Gharib, CIMMYT-Tanzania, Phone # +254 (20) 7224897; e-mail: m.gharib@cgiar.org.

Do you have any questions for me?

**<*Enumerator: pause and respond to any questions raised, then continue with the following statement*>.**

May I continue to ask you some questions? _____Yes _____No ________Initials

By continuing this interview, you indicate your willingness to **voluntarily** participate in the study.

# Module A1. Survey Staff Details

***Enumerator: Please complete A01, A02, A07-A12 before you approach the HH head for the interview. Be sure to complete X01_a at the time you start the interview***

A01. Date of interview (Day/month/year): ..../....../......... A02. Interviewed by:.........................................................

A03. Date checked (Day/month/year): ..../....../......... A04. Checked by:..................................................................

A05. Date entered (Day/month/year): ..../....../......... A06. Entered by:..................................................................

| X01. ***Enumerator: Please write the time you START the interview (hh:mm)*** | X01_a (hh): | X01_b (mm): |
| --- | --- | --- |

# Module A2. Household Identification

| No**.** | Question **(*Instructions*)** | Response | | | Code |
| --- | --- | --- | --- | --- | --- |
| A07 | Country | Tanzania | | |  |
| A07_1 | Region |  | | |  |
| A08 | District |  | | |  |
| A11 | Parish/Ward |  | | |  |
| A12 | Village |  | | |  |
| A13 | Name of the Household Head |  | | |  |
| A14 | Phone number of the Household Head |  | | |  |
| A15 | Latitude of the dwelling unit (in decimal degrees) ***(Insert North or South in first cell)*** |  | . | |  |
| A16 | Longitude of the dwelling unit (in decimal degrees) | E. | . | |  |
| A17 | Altitude of the dwelling unit (meters above sea level) | meters | | |  |
| A18 | GPS device ID number |  | | |  |
| A19 | Entry number for A15-A17 on GPS device |  | | |  |
| A20 | Distance to the nearest main crop market in KM and walking time(minutes)? | KM | | min |  |
| A21 | Transport cost to the main market per person? |  | | |  |
| A22 | Experience (years) in growing maize? |  | | |  |
| A23 | Distance to nearest government agricultural field officer in KM and walking time(minutes)? | KM | | min |  |
| A24 | Transport cost to the nearest government extension office per person? |  | | |  |
| A25 | No. of contacts with government extension in 2018 on maize? |  | | |  |
| A26 | Number of years a farmer has lived in the area? |  | | |  |
| A27 | Distance to the nearest financial institution? | Km | | |  |
| A29 | Have you attended any demonstration farm in your village? | 1. No | | |  |
|  |  | 1. Yes | | |  |
| A30 | Have you attended any demonstration farm in other villages? | 1. No | | |  |
|  |  | 1. Yes | | |  |

# Module B. Household Roster

***Enumerator read ALL:*** I will start by asking you a few questions about your HH members, such as their names, ages, and sex.

B00. Can you first tell me the TOTAL number of people that are in your household? By household I mean the people related or unrelated, who live together at your house/compound and make common provision for food. They regularly take all their food from the same pot, and/or share the same grain store, or pool their incomes for the purposes of purchasing food. ***(Write response in space provided below.)***

B00_a. __________________household members…

| B01  Member ID code   1. Head 2. Spouse | B02  Now please list the names of the household head and spouse ONLY.  ***(Write the name of the head first, followed by the spouse. If more than one spouse, write the name of the spouse that is MOST INVOLVED IN MAIZE FARMING.)*** | B03  What is [NAME]’s gender?   1. Female 2. Male | B04  How many months since October 2017 was [NAME] away from the household? | B05  What is the age of [NAME]? (years) | B06  Years of farming experience | B07  How many years of formal education did [NAME] complete?  ***(Enter 0 if no formal education)*** | B08  What is the main occupation of [NAME]?  ***(Use code B08)*** | B09  What is [NAME’s] marital status?   1. Monogamous married or non-formal union 2. Polygamous married or non-formal union 3. Separated 4. Divorced 5. Widowed 6. Never married |
| --- | --- | --- | --- | --- | --- | --- | --- | --- |
| 1 |  |  | mos | yrs |  | yrs |  |  |
| 2 |  |  | mos | yrs |  | yrs |  |  |

| Occupation **Code B08** | | | | | |
| --- | --- | --- | --- | --- | --- |
| Agriculture self-employed  Agriculture wage labour  Non-agric. self-employment  Non-agric. wage labour  Salaried worker  Student  Unemployed  Retired  Too young to work  99. Other, specify…… |  |  |  |  |  |

**Module B. Household Roster (continued)**

***Enumerator read:*** Now we have a few questions about ALL HOUSEHOLD MEMBER, NOT INCLUDING THE HOUSEHOLD HEAD OR SPOUSE.

| B01  Member ID code | B02  Now please list the names of all household members, if there are any.  ***(check the response given to B00_a.)*** | B03  What is [NAME]’s gender?   1. Female 2. Male | B05  What is the age of [NAME]? (years) | B10  What is [NAME]’s relationship to the household head?  ***(Use code B10)*** | B11  Does [NAME] currently attend school?   1. No 2. Yes   ***(Read note B11)*** | B12  Who manages the school [NAME] attends?   1. Government 2. Private 3. NGO/religious organization   ***(Ask this if B11 is yes)*** |
| --- | --- | --- | --- | --- | --- | --- |
| 3 |  |  | yrs |  |  |  |
| 4 |  |  | yrs |  |  |  |
| 5 |  |  | yrs |  |  |  |
| 6 |  |  | yrs |  |  |  |
| 7 |  |  | yrs |  |  |  |
| 8 |  |  | yrs |  |  |  |
| 9 |  |  | yrs |  |  |  |
| 10 |  |  | yrs |  |  |  |
| 11 |  |  | yrs |  |  |  |
| 12 |  |  | yrs |  |  |  |
| Relation **Code B10** | | | **Note B11** | | | |
| 1. Son/daughter 2. Sister/brother 3. Niece/nephew 4. Other (specify) | | | ***Enumerators read:*** Currently attending school refers to primary, secondary, vocational/technical, or professional school. Students are counted as attending even if they are not attending because they are on holiday, on vacation, studying for exams, sick, or if school is temporarily closed. | | | |

# Module C. Farm Plot Characteristics 2017/2018

***Enumerator read:*** We would now like to collect some information about the farm plots your HH cultivated in the 2018 major and the 2017 minor season. A plot is a continuous piece of land on which a unique crop or mixture of crops is grown under a uniform, consistent crop management system. A plot is continuous and should not be split by a path > 1-meter width. Plot boundaries are defined according to the crops grown and the operator.

C00. Total farm size owned by household ______ acres

| C01  Please list all plots your HH cultivated in the 2017 minor season and the 2018 major season (***include plots rented in or borrowed)***  Plot ID | | C02  Plot/location name (as called by farmer) | C03  Which crops did you grow on this plot in the 2017 minor season?  ***(Use code C03)*** | | | | | | | | C04  Which crops did you grow on this plot in the 2018 major season?  ***(Use code C03)*** | | | | | | | C05  What is the area of [PLOT NAME]? |
| --- | --- | --- | --- | --- | --- | --- | --- | --- | --- | --- | --- | --- | --- | --- | --- | --- | --- | --- |
|  |  |  |  |  |  |  |  |  |  |  |  |  |  |  |  |  |  | Farmer Estimate  ***(in acres)*** |
|  |  |  | C03_a | | C03_b | | C03_c | C03_d | | C03_e | C04_a | | C04_b | C04_c | | C04_d | C04_e |  |
| **1** | |  |  | |  | |  |  | |  |  | |  |  | |  |  |  |
| **2** | |  |  | |  | |  |  | |  |  | |  |  | |  |  |  |
| **3** | |  |  | |  | |  |  | |  |  | |  |  | |  |  |  |
| **4** | |  |  | |  | |  |  | |  |  | |  |  | |  |  |  |
| **5** | |  |  | |  | |  |  | |  |  | |  |  | |  |  |  |
| **6** | |  |  | |  | |  |  | |  |  | |  |  | |  |  |  |
| **7** | |  |  | |  | |  |  | |  |  | |  |  | |  |  |  |
| **8** | |  |  | |  | |  |  | |  |  | |  |  | |  |  |  |
| **9** | |  |  | |  | |  |  | |  |  | |  |  | |  |  |  |
| **10** | |  |  | |  | |  |  | |  |  | |  |  | |  |  |  |
| **11** | |  |  | |  | |  |  | |  |  | |  |  | |  |  |  |
| **12** | |  |  | |  | |  |  | |  |  | |  |  | |  |  |  |
| Crops **Code C03**   1. Maize 2. Paddy 3. Sorghum 4. Wheat 5. Millets | 1. Cassava 2. Sweet potatoes 3. Round potatoes 4. Yams | | | 1. Coco yams 2. Beans 3. Chick peas 4. Cow peas 5. Bambaranuts | | 1. Field peas 2. Pigeon peas 3. Groundnuts 4. Sunflower 5. Simsim 6. Oil Palm | | | 1. Soya beans 2. Castor seeds 3. Tomatoes 4. Cabbage 5. Onions 6. Amaranth’s | | | 1. Chillies 2. Water melon 3. Cotton 4. Coffee 5. Cashew nuts 6. Tobacco 7. Sisal | | | 1. Tea 2. Pyrethrum 3. Sugarcane 4. Palm oil 5. Flowers   99. Other (specify)________ | | | |

#

dd

# Module J. Livestock, Farm Tools, and Land Rental 2017/2018

***Enumerator read:*** These questions are about your household’s rental of oxen, farm tools, transport, or land for maize farming in the 2018 major and 2017 minor seasons.

| J01  Asset type | | J02  Did your household hire [ASSET] for maize production during the 2018 major season?   1. No 2. Yes | J03  If ‘yes’ to J02, how much in total did you pay for hiring [ASSET] during the 2018 major season? | J04  If ‘yes’ to J02, for maize production on which plots was [ASSET] hired for the 2018 major season?  ***(Verify that the plot names the farmer provides are consistent with those listed on the previous page, then enter plot ID)*** | | | J05  Did your household hire [ASSET] for maize production during the 2017 minor season?   1. No 2. Yes | J06  If ‘yes’ to J05, how much in total did you pay for hiring [ASSET] during the 2017 minor season? | J07  If ‘yes’ to J05, for maize production on which plots was [ASSET] hired for the 2017 minor season?  ***(Verify that the plot names the farmer provides are consistent with those listed on the previous page, then enter plot ID)*** | | |
| --- | --- | --- | --- | --- | --- | --- | --- | --- | --- | --- | --- |
| J01_a  Asset Code | J01_b  Asset name |  |  | J04_a  Plot ID #1 | J04_b  Plot ID #2 | J04_c  Plot ID #3 |  |  | J07_a  Plot ID #1 | J07_b  Plot ID #2 | J07_c  Plot ID #3 |
| 1 | Oxen |  |  |  |  |  |  |  |  |  |  |
| 2 | Sprayer |  |  |  |  |  |  |  |  |  |  |
| 3 | Slasher |  |  |  |  |  |  |  |  |  |  |
| 4 | Hoe |  |  |  |  |  |  |  |  |  |  |
| 5 | Tarpaulin |  |  |  |  |  |  |  |  |  |  |
| 6 | Thresher |  |  |  |  |  |  |  |  |  |  |
| 7 | Wheelbarrow |  |  |  |  |  |  |  |  |  |  |
| 8 | Ox cart |  |  |  |  |  |  |  |  |  |  |
| 9 | Ox plough |  |  |  |  |  |  |  |  |  |  |
| 10 | Bicycle |  |  |  |  |  |  |  |  |  |  |
| 11 | Motorcycle |  |  |  |  |  |  |  |  |  |  |
| 12 | Motor vehicle |  |  |  |  |  |  |  |  |  |  |
| 13 | Land |  |  |  |  |  |  |  |  |  |  |
| 14 | Tractor |  |  |  |  |  |  |  |  |  |  |
| 15 | Power tillers |  |  |  |  |  |  |  |  |  |  |

# Module K: Drought Risk Perceptions and Adjustments

***Enumerator read:*** We will start our conversation by asking a few questions about weather in your area, especially rainfall and dry spells.

| No. | Question ***(Instructions)*** | | Response | | | | | Code/Rank | |
| --- | --- | --- | --- | --- | --- | --- | --- | --- | --- |
| K01 | Do you regularly obtain information on expected rainfall and temperature from any source? | | No ***(skip to K03)*** | | | | | 0 | |
|  |  |  | Yes ***(continue to K02)*** | | | | | 1 | |
| K02 | From which source(s) do you obtain information on expected rainfall and temperature? ***(multiple responses are possible, circle all that the respondent mentions)*** | | Government | | | | | 1 | |
|  |  |  | Radio/TV | | | | | 2 | |
|  |  |  | Fellow farmer | | | | | 3 | |
|  |  |  | NGO | | | | | 4 | |
|  |  |  | Other, specify…….. | | | | | 99 | |
|  | | | **2018** | | **2017** | **2016** | **2015** | | **2014** |
| K03 | In which of the last five years did your household experience dry spells during the major rainy season of the year?   1. No 1. Yes   ***(If response is 0 for the year, skip to the next year, i.e. don’t ask K04-K05 if K03=0.)*** | |  | |  |  |  | |  |
| K04 | In which of the last five years did dry spells during the major rainy season lead to substantial harvest loss for maize?   1. No 1. Yes | |  | |  |  |  | |  |
| K05 | What changes, if any, has your HH made to help reduce the negative impacts of drought? ***(Use codes K05) (Use comma to separate multiple responses, where given)*** | |  | | | | | | |
| K06 | Over the last ten years how many times was your household affected negatively by drought? | | **________**_Times | | | | | | |
| Adjustment mechanisms: **Code K05** | | | |  | | | | | |
| 1. None 2. Grow drought tolerant maize varieties 3. Grow early maturing crops 4. Income diversification (e.g., find off-farm income sources) 5. Crop diversification (more crops on farm or intercropping) 6. Early planting 7. Irrigation | | 1. Shift farming to watlands 2. Minimum tillage 3. Stone and soil bunds/terracing 4. Precautionary savings 5. Selling assets, e.g. livestock 6. Borrow money 7. Other, specify ………………………….. | | | | | | | |

**Module M. Social Capital & Market Access**

***Enumerator read:*** Now I would like to talk with you about different groups in your village that you belong to, like farmers’ groups, religious groups, and so on.

| M01 In the last 3 years, were you a member of any of the following groups? | | |
| --- | --- | --- |
| M01_a - Code | M01_b - Group name | M01_c 0. No 1. Yes |
| 1 | Input supply/farm coop |  |
| 2 | Crop/seed producer & marketing group |  |
| 3 | Farmer association |  |
| 4 | Farmer research group |  |
| 5 | Women’s association |  |
| 6 | Religious congregation |  |
| 7 | Social club |  |
| 8 | Saving and credit group |  |
| 9 | Merry-go-round group |  |
| 10 | Local administration |  |
| 11 | Burial group |  |
| 12 | Communal labor group |  |
| 99 | Other, specify |  |

# Module N. Access to Information and Credit

***Enumerator read:*** On this page I have some questions about where you get information on improved maize varieties/crops and about your sources of credit to purchase maize/crop inputs.

| No. | Question **(*Instructions*)** | | | Response | | | Code |
| --- | --- | --- | --- | --- | --- | --- | --- |
| N01 | In the 2018 major and 2017 minor season, did you receive any information on new varieties of maize seed? | | | No ***(Skip to N04)*** | | | 0 |
|  |  |  |  | Yes ***(Continue to N02)*** | | | 1 |
| N02 | From what sources did you receive information about new maize seed?  ***(Record up to three in order of importance.)***  ***(Use N02 codes)*** | | | Most important information source | | |  |
|  |  |  |  | 2^nd^ most important information sour | | |  |
|  |  |  |  | 3^rd^ most important information source | | |  |
| N02_a | On average, how useful was the information received from the first most important source? Would you say the information received was useless, not very useful, useful, or very useful? | | | Useless | | | 1 |
|  |  |  |  | Not very useful | | | 2 |
|  |  |  |  | Useful | | | 3 |
|  |  |  |  | Very useful | | | 4 |
| N02_b | On average, how useful was the information received from the 2^nd^ most important source? Would you say the information received was useless, not very useful, useful, or very useful? | | | Useless | | | 1 |
|  |  |  |  | Not very useful | | | 2 |
|  |  |  |  | Useful | | | 3 |
|  |  |  |  | Very useful | | | 4 |
| N02_c | On average, how useful was the information received from the 3^rd^ most important source? Would you say the information received was useless, not very useful, useful, or very useful? | | | Useless | | | 1 |
|  |  |  |  | Not very useful | | | 2 |
|  |  |  |  | Useful | | | 3 |
|  |  |  |  | Very useful | | | 4 |
| N03 | Which source do you trust the most?  ***(Use N02 codes)*** | | |  | | |  |
| N04 | If you need to borrow money to buy fertilizer, seed, and other inputs for maize production how likely is it that you will be able to borrow money from your likely sources of credit? | | | Extremely likely (about 100% chance) | | | 1 |
|  |  |  |  | Quite likely (about 75%) | | | 2 |
|  |  |  |  | Neither likely or unlikely (about 50%) | | | 3 |
|  |  |  |  | Quite unlikely (about 25%) | | | 4 |
|  |  |  |  | Extremely unlikely (about 0%) | | | 5 |
| N05 | Who is your main source of credit ***(use codes N05)*** | | |  | | |  |
| N06 | Which of the following is true? Your household’s current income (2017/2018)  [Read the possible responses at right] | | | Allows you to build your savings | | | 1 |
|  |  |  |  | Allows you to save a little | | | 2 |
|  |  |  |  | Income = expenses | | | 3 |
|  |  |  |  | Is not sufficient, so you need to use your savings to meet expenses | | | 4 |
|  |  |  |  | Is really not sufficient, so you need to borrow to meet expenses | | | 5 |
| N07_A | By what mode of transport do you usually travel to the place where you buy hybrid and OPV maize seed in bags?  ***(Circle only the main mode of transport)(ask N07_B if only N07 is 98)*** | | | Foot | | | 1 |
|  |  |  |  | Bicycle | | | 2 |
|  |  |  |  | Vehicle | | | 3 |
|  |  |  |  | Bodaboda/Daladala | | | 4 |
|  |  |  |  | Other | | | 97 |
|  |  |  |  | Not applicable | | | 98 |
| Information source **Code N02** | | | | | Credit source **Code N05** | | |
| 1. Government extension 2. Pvt. agric ext. svc./company 3. NGO 4. Farmer field day/agricultural show 5. Demonstration plot | | | 1. Input shop supplier 2. Other farmers 3. Electronic media (TV, radio) 4. Paper media   99. Other (specify …………………………) | | 1. Bank  2. Rural microfinance institution  3. Village savings and loan association  4. Traders 5. Money lenders  6. Family members/relatives 7. Friend  99. Other (specify)………………………… | | |
| N07_B | | If you don’t use improved seed currently what would be the nearest market to purchase improved seed (OPV, Hybrids) from? | | KM | | | |
| N08 | | How long does it take, in minutes, to travel (one way) to the nearest place to buy hybrid and OPV maize seed in bags | | Minutes  (one-way) | |  | |

# Module O. Food Security

***Enumerator read:*** The interview is almost over, thanks for your patience. Now I have some questions about the food security situation of your household in the last four weeks, that is between [DATE four weeks earlier] and [the DATE yesterday].

| No. | Question **(*Instructions*)** | Response | Code | |  |
| --- | --- | --- | --- | --- | --- |
| O01 | In the past four weeks, did you worry that your household would not have enough food? | No ***(Skip to O03)*** | 0 | |  |
|  |  | Yes ***(Continue to O02)*** | 1 | |  |
| O02 | How often did this happen? | Rarely (once or twice in the past 4 wks) | 1 | |  |
|  |  | Sometimes (3 to 10 times in the past 4 wks) | 2 | |  |
|  |  | Often (more than 10 times in the past 4 wks) | 3 | |  |
| O03 | In the past four weeks, were you or any household member not able to eat the kinds of food you preferred because of a lack of resources? | No ***(Skip to O05)*** | 0 | |  |
|  |  | Yes ***(Continue to O04)*** | 1 | |  |
| O04 | How often did this happen? | Rarely (once or twice in the past 4 wks) | 1 | |  |
|  |  | Sometimes (3 to 10 times in the past 4 wks) | 2 | |  |
|  |  | Often (more than 10 times in the past 4 wks) | 3 | |  |
| O05 | In the past four weeks, did you or any household member have to eat a limited variety of food due to a lack of resources? | No ***(Skip to O07)*** | 0 | |  |
|  |  | Yes ***(Continue to O06)*** | 1 | |  |
| O06 | How often did this happen? | Rarely (once or twice in the past 4 wks) | 1 | |  |
|  |  | Sometimes (3 to 10 times in the past 4 wks) | 2 | |  |
|  |  | Often (more than 10 times in the past 4 wks) | 3 | |  |
| O07 | In the past four weeks, did you or any household member have to eat some foods that you really did not want to eat because of a lack of resources to obtain other types of food? | No ***(Skip to O09)*** | 0 | |  |
|  |  | Yes ***(Continue to O08)*** | 1 | |  |
| O08 | How often did this happen? | Rarely (once or twice in the past 4 wks) | 1 | |  |
|  |  | Sometimes (3 to 10 times in the past 4 wks) | 2 | |  |
|  |  | Often (more than 10 times in the past 4 wks) | 3 | |  |
| O09 | In the past four weeks, did you or any household member have to eat a smaller meal than you felt you needed because there was not enough food? | No ***(Skip to O11)*** | 0 | |  |
|  |  | Yes ***(Continue to O10)*** | 1 | |  |
| No. | Question **(*Instructions*)** | Response | Code | |  |
| O10 | How often did this happen? | Rarely (once or twice in the past 4 wks) | 1 | |  |
|  |  | Sometimes (3 to 10 times in the past 4 wks) | 2 | |  |
|  |  | Often (more than 10 times in the past 4 wks) | 3 | |  |
| O11 | In the past four weeks, did you or any other household member have to eat fewer meals in a day because there was not enough food? | No ***(Skip to O13)*** | 0 | |  |
|  |  | Yes ***(Continue to O12)*** | 1 | |  |
| O12 | How often did this happen? | Rarely (once or twice in the past 4 wks) | 1 | |  |
|  |  | Sometimes (3 to 10 times in the past 4 wks) | 2 | |  |
|  |  | Often (more than 10 times in the past 4 wks) | 3 | |  |
| O13 | In the past four weeks, was there ever no food to eat of any kind in your household because of lack of resources to get food? | No ***(Skip to O15)*** | 0 | |  |
|  |  | Yes ***(Continue to O14)*** | 1 | |  |
| O14 | How often did this happen? | Rarely (once or twice in the past 4 wks) | 1 | |  |
|  |  | Sometimes (3 to 10 times in the past 4 wks) | 2 | |  |
|  |  | Often (more than 10 times in the past 4 wks) | 3 | |  |
| O15 | In the past four weeks, did you or any household member go to sleep at night hungry because there was not enough food? | No ***(Skip to O17)*** | 0 | |  |
|  |  | Yes ***(Continue to O16)*** | 1 | |  |
| O16 | How often did this happen? | Rarely (once or twice in the past 4 wks) | 1 | |  |
|  |  | Sometimes (3 to 10 times in the past 4 wks) | 2 | |  |
|  |  | Often (more than 10 times in the past 4 wks) | 3 | |  |
| O17 | In the past four weeks, did you or any household member go a whole day and night without eating anything because there was not enough food? | No ***(end the interview)*** | 0 | |  |
|  |  | Yes ***(Continue to O18)*** | 1 | |  |
| O18 | How often did this happen? | Rarely (once or twice in the past 4 wks) | 1 | |  |
|  |  | Sometimes (3 to 10 times in the past 4 wks) | 2 | |  |
|  |  | Often (more than 10 times in the past 4 wks) | 3 | |  |
| O19 | Was your household self-sufficient in maize last year (i.e. from Sep 2017 to Aug 2018)? | No ***(Continue to O20)*** | 0 | |  |
|  |  | Yes ***(skip to O21)*** | 1 | |  |
| O20 | From Sep 2017 to Aug 2018, how many months dis your household NOT have maize from its own production? | months |  | |  |
| 021 | In which months did your household eat its own maize produce? (***Use code F05_A)*** |  | |  | |
| 022 | Did your household purchase any maize grain, packaged sifted maize meal, and posho meal in the last 12 months (Aug 2017 to July 2018)? | No | | 0 | |
|  |  | Yes | | 1 | |
| 023 | Did your household purchase any green maize in the last 12 months (Aug 2017 to July 2018)?  ***(Enumerator: If O21 and O22 are No skip to next section)*** | No | | 0 | |
|  |  | Yes | | 1 | |
